# Highly Segregated Biocomposite Membrane as a Functionally Graded Template for Periodontal Tissue Regeneration

**DOI:** 10.3390/membranes11090667

**Published:** 2021-08-30

**Authors:** Syed Saad B. Qasim, Mirza Rustum Baig, Jukka Pekka Matinlinna, Umer Daood, Adel Al-Asfour

**Affiliations:** 1 Department of Bioclinical Sciences, Faculty of Dentistry, Kuwait University, P.O. Box 24923, Safat 13110, Kuwait; 2 Department of Restorative Sciences, Faculty of Dentistry, Kuwait University, P.O. Box 24923, Safat 13110, Kuwait; mirza.baig@ku.edu.kw; 3 Dental Materials Science, Applied Oral Sciences, Faculty of Dentistry, The University of Hong Kong, Hospital Road 34, Sai Ying Pun, Hong Kong, China; jpmat@hku.hk; 4 Division of Dentistry, School of Medical Sciences, University of Manchester, Manchester M13 9PL, UK; 5 Clinical Dentistry, Restorative Division, Faculty of Dentistry, International Medical University Kuala Lumpur, 126, Jalan Jalil Perkasa 19, Bukit Jalil, Kuala Lumpur 57000, Malaysia; umerdaood@imu.edu.my; 6 Department of Surgical Sciences, Faculty of Dentistry, Kuwait University, P.O. Box 24923, Safat 13110, Kuwait; adel.alasfour@ku.edu.kw

**Keywords:** chitosan, hydroxyapatite, guided tissue regeneration, periodontal engineering

## Abstract

Guided tissue regeneration (GTR) membranes are used for treating chronic periodontal lesions with the aim of regenerating lost periodontal attachment. Spatially designed functionally graded bioactive membranes with surface core layers have been proposed as the next generation of GTR membranes. Composite formulations of biopolymer and bioceramic have the potential to meet these criteria. Chitosan has emerged as a well-known biopolymer for use in tissue engineering applications due to its properties of degradation, cytotoxicity and antimicrobial nature. Hydroxyapatite is an essential component of the mineral phase of bone. This study developed a GTR membrane with an ideal chitosan to hydroxyapatite ratio with adequate molecular weight. Membranes were fabricated using solvent casting with low and medium molecular weights of chitosan. They were rigorously characterised with scanning electron microscopy, Fourier transform infrared spectroscopy in conjunction with photoacoustic sampling accessory (FTIR-PAS), swelling ratio, degradation profile, mechanical tensile testing and cytotoxicity using human osteosarcoma and mesenchymal progenitor cells. Scanning electron microscopy showed two different features with 70% HA at the bottom surface packed tightly together, with high distinction of CH from HA. FTIR showed distinct chitosan dominance on top and hydroxyapatite on the bottom surface. Membranes with medium molecular weight showed higher swelling and longer degradation profile as compared to low molecular weight. Cytotoxicity results indicated that the low molecular weight membrane with 30% chitosan and 70% hydroxyapatite showed higher viability with time. Results suggest that this highly segregated bilayer membrane shows promising potential to be adapted as a surface layer whilst constructing a functionally graded GTR membrane on its own and for other biomedical applications.

## 1. Introduction

Chronic periodontitis is a chronic-remitting inflammatory disease of periodontal tissues displaying episodes of inflammation and immune suppression resulting in periodontal destruction and ultimately tooth loss [1]. The pivotal functions of currently available periodontal treatments are the control of existing infection, resolution of inflammation, arresting disease progression and prevention of recurrence of disease [2]. During the past three decades, numerous treatment approaches involving surgical and non-surgical techniques and regenerative procedures have been used [2,3]. Whilst these techniques lead to improvements in the clinical condition, healing largely occurs by repair, with the formation of a long junctional epithelium, fibrosis, bone remodelling and very limited regeneration of periodontal attachment [2,4]. True regeneration of diseased periodontal tissues is a complex process [5], which is characterised by formation of root cementum and functionally oriented periodontal ligaments (PDL) inserted into the alveolar bone [2].

Guided tissue regeneration (GTR) membrane when used to treat periodontal defects has shown some promising results [6] and is based on a solid biological principle [7]. The rational is to use a physical barrier to selectively guide cellular proliferation by restricting access of epithelial cells, gingival connective tissue and cells that lead to fibrosis invading the lesion, to allow population with cells having a potential for the regeneration of lost attachment [8]. Natural and synthetic polymers have been utilised to fabricate a number of different GTR membranes. Biodegradable membranes are preferred over non-degradable counterparts, to avoid second stage surgery for removal [9,10]. The use of GTR membranes can result in successful regeneration of periodontium, although there are certain limitations in using these techniques and the clinical outcomes are still unpredictable. This unpredictability could be due to non-uniform degradation profiles and limited bioactivity. Recently, researchers have proposed that a multi-layered bioactive membrane could serve as the next generation of GTR membranes [6]. Therefore, it is important to improve these biomaterial concepts to achieve predictable clinical outcomes.

Chitosan (CH) is a partially deacetylated form of chitin which is a naturally derived amino polysaccharide [11,12]. It is a copolymer of glucosamine and N-acetylglucosamine, and has a number of favourable properties such as biocompatibility, biodegradability and an antimicrobial nature, which has resulted in wide interest for its use in various biomedical applications, including tissue engineering and regenerative medicine [13]. CH has minimal host immune rejection [14], forming cationic clusters [15] that can also instil nerve regeneration, bone repair and wound healing [16]. A major advantage of the CH compound is its ability to act as a substrate for bone tissue regeneration resembling glycosaminoglycans found as principal components inside the bone extracellular matrix [17]. Moreover, CH can also be reinforced with finely divided osteogenic mineral phases to improve mechanical strength and stimulate bone tissue formation [18].

Synthetic Hydroxyapatite (HA) is well known due to its excellent biocompatibility and bioactivity. It has chemical similarity to the inorganic bone matrix and has the potential to form composite formulations of CH with HA which have also shown promising results in the past. CH:HA composites have been studied previously by various groups [19,20,21,22]. In our previous studies, we have reported detailed characterisation of a freeze gelated core layer of CH and HA, along with electrospun fibres in random and aligned orientations with the potential of becoming a critical component of functionally graded membrane with structural gradients to meet the local functional requirements of the native tissue structure and function [20,23,24]. The aim of the current study was to fabricate and rigorously characterise a nonporous CH:HA membrane to function as a surface layer of a trilayered GTR membrane to face the defect side.

## 2. Materials and Methods

### 2.1. Materials

Two different Mw chitosans (CH) were used, of medium molecular weight (MMw) (Mol. Wt. 190–310 KDa) and low molecular weight (LMw) (Mol. Wt. 50 to 190 KDa), both with a degree of deacetylation (DD) of 75–85% (Sigma Aldrich, Gillingham, UK). Hydroxyapatite (HA) powder used was obtained from Plasma Biotal, UK. The HA used has a particle size of 0.5 ± 2 to 12 ± 1 μm (Malvern Mastersizer 3000^®^ Hydro EV, Malvern Instruments Ltd., Worcestershire, UK). Alpha Minimum essential medium (α-MEM) (Lonza, Verviers, Belgium), Dulbecco’s modified Eagle’s medium (DMEM) (Biosera, Ringmer, UK) and Foetal Calf Serum (FBS) were obtained from GIBCO, Invitrogen Corporation, Loughborough, UK. Acetic acid and hen egg lysozyme were acquired from Sigma Aldrich, Gillingham, UK.

### 2.2. Fabrication of CH:HA Membranes

Simple solvent casting technique was adapted to prepare neat CH and composite membranes. Plain membranes were prepared at a concentration (conc) of 2 wt.%. MMw and LMw CH were dissolved in 0.2 M acetic acid by heating to 50 °C under constant stirring for an hour (h). Glycerol was also added to the solution at a conc of 0.25 mL/gm of the polymer used. After 24 h of mixing at room temperature (RT) 20 ± 2 °C, a homogenous solution was formed. Desired volumes of these solutions (30 mL) were taken up from the centrifuged solutions and casted in polytetrafluoroethylene (PTFE) petri dishes.

Composite membranes were prepared with neat solutions of LMw and MMw in a similar procedure as described above. After the addition of glycerol, HA was added in ratios of 30:70, 50:50 and 70:30, followed by constant stirring at room temperature (RT). These solutions were mixed for 24 h and then desired volumes (30 mL) were casted in plastic petri dishes. In order to achieve a high segregation of the HA and CH, the petri dish lid was left on for 48 h for controlled solvent evaporation and then removed for rapid solvent evaporation. All membranes were neutralised with 1 M NaOH/50% (1:1) ethanol solution in distilled water (Di) for an hour and rinsed with phosphate buffered saline thrice for 15 min.

### 2.3. Scanning Electron Microscope (SEM) Analysis

Surface characterisation of the CH membranes was conducted by scanning electron microscopy (Philips X-L 20 SEM, Tech Solutions Inc., North Billerica, MA, USA) (spot size: 3.0, with a voltage range of 5–10 kV). The samples were sputter coated under vacuum with carbon and then attached onto aluminium stubs with adhesive carbon dots (Agar Scientific, Essex, UK).

### 2.4. Fourier Transform Infrared (FTIR) Spectroscopy

FTIR spectra of the membranes were acquired by using a Thermo Scientific FTIR Nicolet™ iS™ 50R (Thermo Fisher Scientific Inc., Walhtham, MA, USA) spectrophotometer along with a Photo acoustic sampling (PAS) (MTec, Ames, IA, USA) cell. The mid infrared region (4000–400 cm^−1^) was selected at a resolution 8 cm^−1^ by collecting 128 scans. The PAS cell chamber was purged with dry helium gas. Spectral data were processed by Omnic 9™ Software [25].

### 2.5. Mechanical Testing

Tests were conducted in conditions that were similar to our previous study [20]. Briefly a Bose ELF 3200™ (ElectroForce, New Castle, DE, USA) was used for mechanically testing the membranes (20 mm in length and 5 mm in diameter) under tensile condition. Sample testing was performed in both dry and wet conditions whilst using a 22.5 N load. Wet specimens were hydrated using distilled water for a few minutes and then loaded onto the sample holder. From the acquired stress–strain graphs, the point at which the samples break was used to calculate the ultimate tensile strength (UTS) and the strain (%), while the initial linear gradient was taken as the Young’s Modulus (*E*) [20]. Hydrated samples were neutralised and then analysed.

### 2.6. Swelling Ratio and Degradation 

Samples (13 mm Ø) were neutralised, dried and then weighed before storing in PBS at 37 °C with membranes retrieved at pre-set time points of 0, 15 and 30 min and 1, 24 and 48 h. Prior to weighing, all excess water was removed by filter paper sheets. The swelling ratio was calculated using the formula [20]:

Swell Ratio % *(Q)* = (W_w_ − W_d_)/W_d_ × 100(%) (1)

where W_d_ is dry weight and W_w_ is wet weight.

Dried neutralised membranes were weighed (W_o_) and samples were then submerged in degradation solution (lysozyme 5 mg/mL in PBS) with incubation at 37 °C. Time points for assessing the weight changes were 24 h, 7, 14, 21 and 28 days. Fresh media was restored after every 3 days. At each time, interval membranes were rinsed with Di and then dried before weighing them again (W_t_). Degradation was calculated using the formula:

Degradation % = (W_o_ − W_t_)/W_o_ × 100 (2)

### 2.7. Water Contact Angle Measurement

The surface wettability of the highly segregated membranes was evaluated by water contact angle measurements at different points. The measurements were carried out by sessile drop method using Goniometer Model 100-00 (200) UK. A drop of water (3 µL) was added by a syringe at room temperature. In order to minimise the experimental error, three readings were performed for each specimen and their average value was reported. The contact angle of the intersection between the liquid (distilled water) and the solid surface of the membrane was measured from the solid surface thorough the liquid to the liquid/vapour tangent line originating from the terminus of the liquid/solid interface. This was used to measure the degree of wettability, whereas no wetting was considered at a contact angle of 180° and complete wetting occurs at an angle of 0.

### 2.8. Cytotoxicity

Cell studies were performed using a similar protocol as reported in our previous investigations [15,18]. Cells were seeded on the side dominated by HA. Briefly, human osteosarcoma (MG63s) were expanded in DMEM supplemented with 10% FCS, 2 mM L-Glutamine, 100 µg/mL of penicillin and streptomycin. hES-MP cells were cultured in α-MEM supplemented with 10% FCS, 2mM L-Glutamine and 100 µg/mL penicillin/streptomycin. Cells were grown in a humidified incubator at 37 °C with 5% CO_2_ with fresh media renewed after every 3 days. Similar growth protocols were adapted for both cell types except T-75 flasks for human embryonic stem cell-derived mesenchymal progenitor cells (hES-MPs), which were coated for 30 min with 0.1% gelatine before seeding the cells. Prior to seeding, cells were grown to 90% confluence and then detached via trypsin. MG63s were used between passages 60–65 while hES-MPs were used between passages 3–7 [20] Membranes were seeded at a density of 25,000 cells by using a stainless-steel seeding ring (10 mm Ø). The membranes were sterilised by ethanol for 30 min initially. Prior to cell seeding, the membranes were washed with PBS and coated with medium containing 10% FCS for 1 h. Cell free membranes were included as controls [20].

### 2.9. Alamar Blue^®^ Staining

Test was conducted in a manner described previously by Qasim et al. [23]. Briefly, cell seeded specimens were carefully washed with PBS at each time point, and 500 µL of Alamar blue^®^ solution (Sigma Aldrich, Gillingham, UK) (diluted 1:10 with PBS) was added followed by incubation at 37 °C for 4 h. A fluorescence plate reader was used to measure the samples at 570 nm using (Bio-TEK, NorthStar Scientific Ltd., Potton, UK) [26].

### 2.10. Mineralised Matrix Deposition (Calcium/Collagen)

Collagen and calcium staining were performed as described previously. Briefly, media was removed after 14, 21 and 28 days, and samples were rinsed with PBS and then fixed with 3.7% formaldehyde for 30 min. After this, samples were washed with PBS and Sirius red solution (direct red dye 1 mg/mL in saturated picric acid, both Sigma, Gillingham, UK) was added to fully submerge samples leaving them for 18 h under mild shaking (20 rpm). Excess dye was washed with Di and samples were destained for quantitative analysis using a known volume of NaOH 0.2 M and methanol (*v/v*) (1:1) for 15 min. Absorption profile of the extracted solution was measured at 490 nm in a 96-well plate reader [20].

Total calcium deposition by hES-MPs was quantified at day 14, 21 and 28 after seeding. Specimens were fixed (see collagen staining) followed by distilled H_2_O washes and then application of 1% Alizarin red solution (pH 4.1) (Sigma, Gillingham, UK) at 1 mL per sample for 20 min on a platform shaker. The unbound dye was removed with distilled water washes. For quantification, the stain was extracted using a known volume of 5% *v/v* perchloric acid to each well for 30 min. The extracted solution was read for absorbance at 405 nm [23].

### 2.11. Statistical Analysis

Unless stated otherwise, all experiments were conducted in triplicates. All presented data refer to mean ± standard deviation (SD). In order to check for any statistically significant differences, one-way ANOVA was performed, followed by Tukey’s post-hoc test. Results with *p*-values of ≤0.05 (*) were considered statistically significant. All data were analysed using Graphpad Prism 5.0™ software18. 

## 3. Results

### 3.1. Optical Images and SEM Analysis

**Figure 1:**
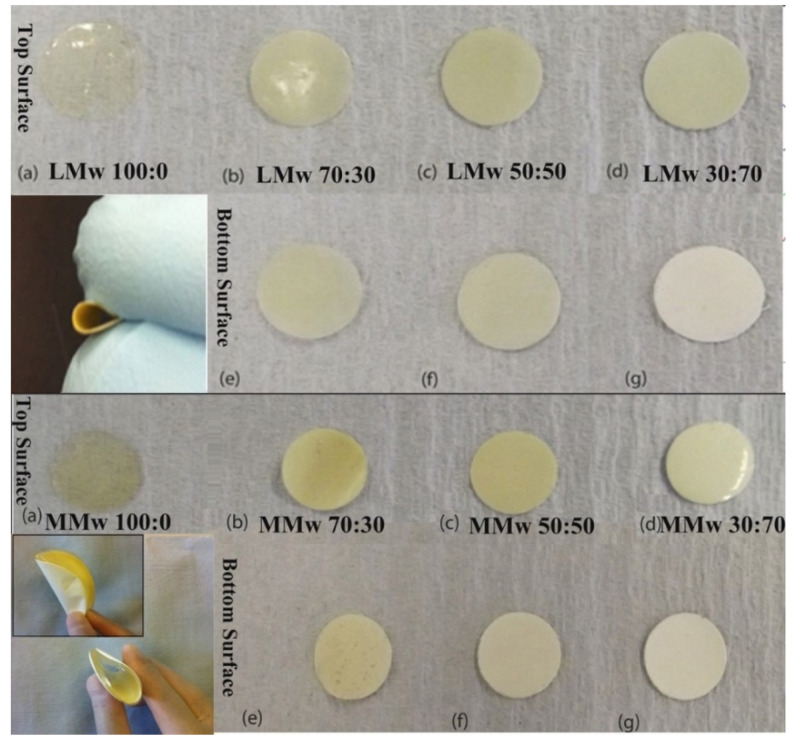
Optical images of LMw and MMwCH:HA membranes in different ratios of CH to HA with clear distinction between top and bottom surfaces of both molecular weight variants. Images taken with a DSLR S5600. The smaller inset image depicts the change in colour in between the top and bottom surface and their ability of the membranes to be bent, which is useful when using as a GTR in periodontal defect side. All (**a**–**d**) are top surfaces of LMw and MMw neat and composite membranes. All (**e**–**g**) are bottom surfaces of LMw and MMw composite membranes.

**Figure 2:**
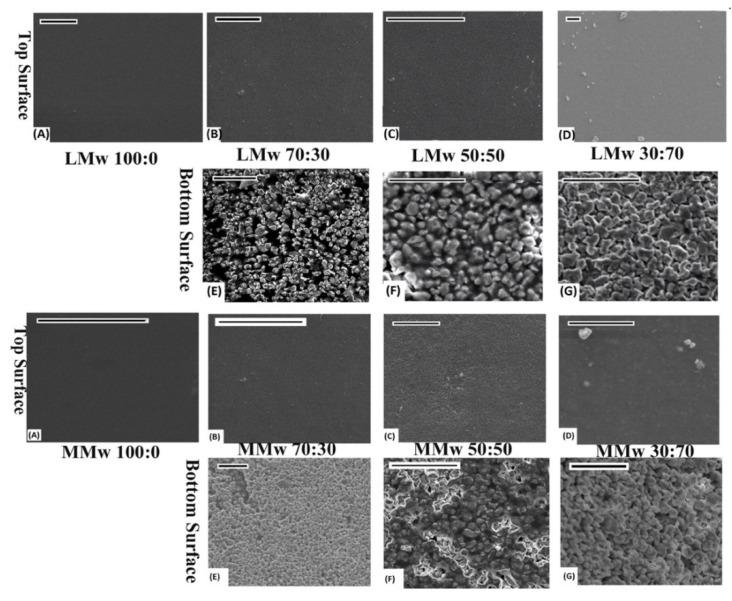
SEM performed on LMW and MMwCH:HA membranes. Top and bottom surface of 100:0, 70:30, 50:50 and 30:70 ratios of CH:HA. All images scaled at 20 µm. (**A**–**D**) are top surfaces of LMw and MMw Neat and composite membranes. (**E**–**G**) are bottom surfaces of composite LMw and MMw membranes.

A clear distinction in features is visible from the optical images. Plain CH membranes are of a translucent nature (see Figure 1). A gradual shift in the colour shade is indicative of the quantity of HA in varying percentages (CH:HA 70:30, 50:50 and 30:70). The 30:70 CH:HA ratios show a completely white surface on the bottom side of the membranes especially with LMw membrane. Upon visual and tactile inspection, the bottom surface displayed slight roughness due to HA and the air interface surface is shiny due to the CH accumulation. The electron micrographs of the neat and composite membranes are shown in Figure 2. At 70% HA, the bottom surface revealed HA particles packed tightly together, and high distinction of CH from HA was observed as the air interface showed very few HA particles. CH dominated the top surface; the only exception was LMw 30:70. This ratio displayed relatively larger particles.

### 3.2. FTIR Spectroscopy

**Figure 3:**
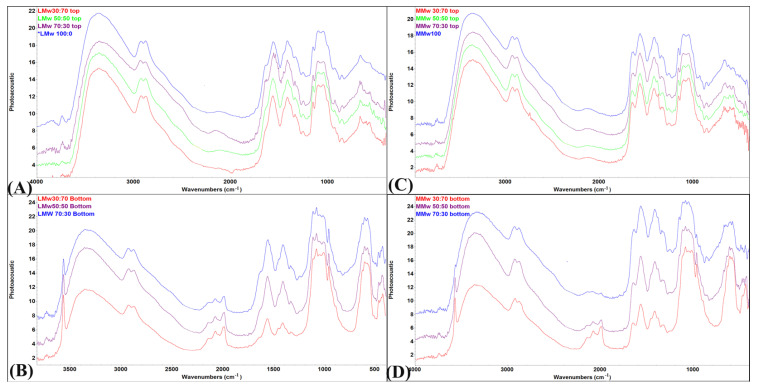
FTIR-PAS spectral profile of (**A**) low molecular weight (LMw) 100:0, 70:30, 50:50 and 30:70 obtained from the top surface; (**B**) LMw 70:30, 50:50 and 30:70 obtained from the bottom surface; (**C**) medium molecular weight (MMw) 100:0, 70:30, 50:50 and 30:70 obtained from the top surface; (**D**) MMw 70:30, 50:50 and 30:70 obtained from the bottom surface.

Spectral data of the neat and composite membranes are shown in Figure 3A–D. The top surface of LMw and MMw displayed higher CH amount as they mimic the spectral profile of plain CH. Few peaks from HA can also be observed on the top surface spectra of both molecular weight variants. In samples with higher ratios of HA (Figure 3B,D), the bands allocated to the mode superimposition of the HA are located in between 1550 to 1700 cm^−1^, −CH amide I and II groups. The functional group of HA gives a typical peak of −OH, that is more exaggerated in the specimens with more HA. Another peak at 3568 cm^−1^ is observed increasing in intensity with higher HA ratios. Bottom spectra deliver more pronounced peaks of different modes of phosphate groups. The existence of CH and its relationship with the phosphate groups of HA can be observed by peculiar broadening of the band at 1050 cm^−1^. Figure 3C,D (top, air interface and bottom surface, petri dish interface) show that the spectral data acquired using MMw CH are comparable to LMw CH:HA. For MMw 70:30, the bottom surface shows more CH dominance in the spectral data. Overall, significant discrepancies can be observed in the top and bottom surface profiles of LMw and MMw with and without HA.

### 3.3. Mechanical Analysis

**Figure 4:**
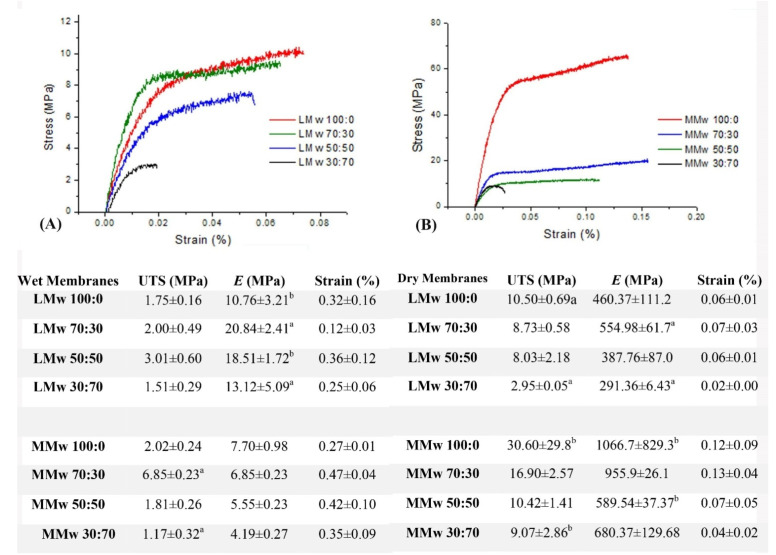
The correlation of stress to strain. (**A**) Representation of the LMw composite membranes in wet conditions, (**B**) representation of the MMw composite membranes in dry conditions. Results of the tensile test are shown in the table, values are a mean ± SD (*n* = 6). Ultimate tensile strength (MPa), Young’s Modulus (*E*) or Elastic Modulus (MPa) and Strain (%). Similar superscripted letters in the same column indicate statistical significance (*p <* 0.05).

Both dry and wet specimens were tested mechanically in tension (Figure 4). During moist conditions, the membranes became weak on handling. Readings showed that neat CH and MMw membranes during dry testing had a greater *E*. The elastic modulus dropped after adding HA, as seen by the decrease in the values with greater ratio. Similarly, in the dry state, it also showed higher E values when compared with neat CH. Results from wet testing of LMw CH and composite membranes showed a greater Young’s modulus when compared with MMw membranes. However, in the dry state, MMw membranes displayed better modulus values. Strain values in most conditions were higher as compared to dry conditions.

### 3.4. Swelling and Weight Loss Analysis

**Figure 5:**
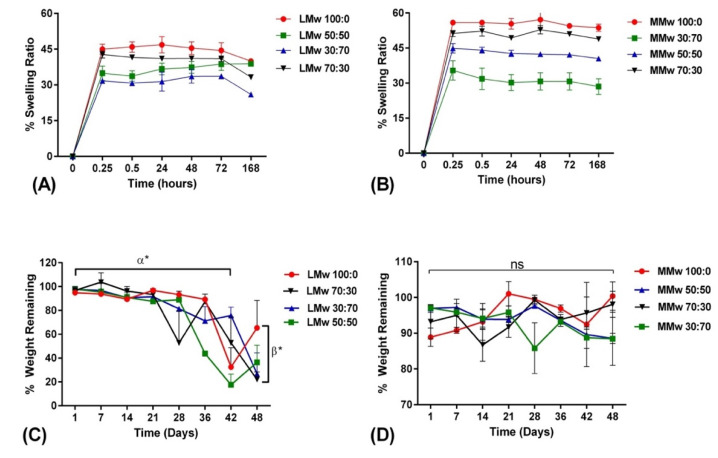
Swelling percentage of (**A**) LMw and (**B**) MMw CH and composite membranes performed over a period of 168 h. Weight remaining percentage of (**C**) LMw and (**D**) MMw composite membranes, performed over an experimental time period of 48 days. Shown values are mean ± SD (*n* = 3).

The swelling ratio of the membranes are shown in Figure 5A,B. Membranes reached an equilibrium after 15 min. Swelling data depict that LMwCH:HA membranes swell less compared to membranes made with MMw. Over the 168 h of swelling profile, the graph achieved a plateau after the 15 min. Swelling tends to decrease as the amount of HA increases. The 30:70 ratios showed least swelling percentage where an overall 30% water uptake is observed after 15 min. Weight remaining studies were performed for up to 48 days (D) on membranes as synthesised with LMw and MMw CH and CH:HA ratios, shown in Figure 5C,D. The LMw CH HA membranes show stability up to 21 days and in between 28 and 48 days, significant percentage weight was observed. This was also observed for LMw 50:50, 30:70 and 70:30, as well. MMw 70:30 follows 100:0 MMw membranes with similar weight remaining. With the addition of HA in MMw membranes an overall 87% weight remains after 48 days. Overall, MMw membranes showed a more stable profile when compared to LMw membranes.

### 3.5. Wetting Contact Angle Analysis

**Figure 6:**
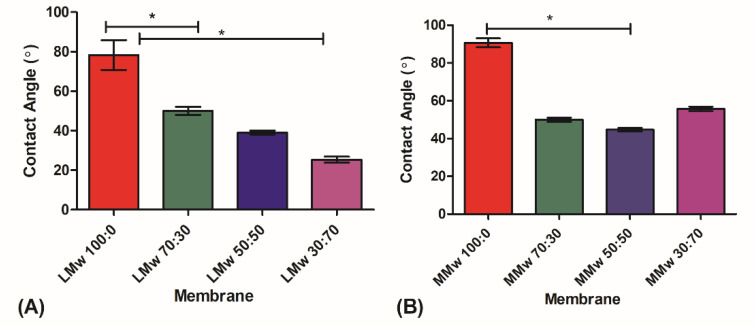
Water contact angle analysis of (**A**) LMw and (**B**) MMw neat and composite membranes 70:30. 50:50 and 30:70. The graphs show mean ± SD (*n* = 3), (*) indicates statistically significant difference. LMw 100:0, 78° ± 6; LMw 70:30, 50°± 1.6; MMw 90° ± 3; MMw 30:70, 55° ± 1.6; MMw 50:50, 45° ± 1.

The wetting contact angle (CA) measurements are shown in Figure 6A,B. The results showed that neat CH LMw and MMw had higher contact angle indicative of hydrophobicity and addition of HA lowered the CA measurement. The lowest values were observed for LMw 30:70 followed by MMw and LMw 50:50, which has almost similar values.

### 3.6. Cytotoxicity Assay

**Figure 7:**
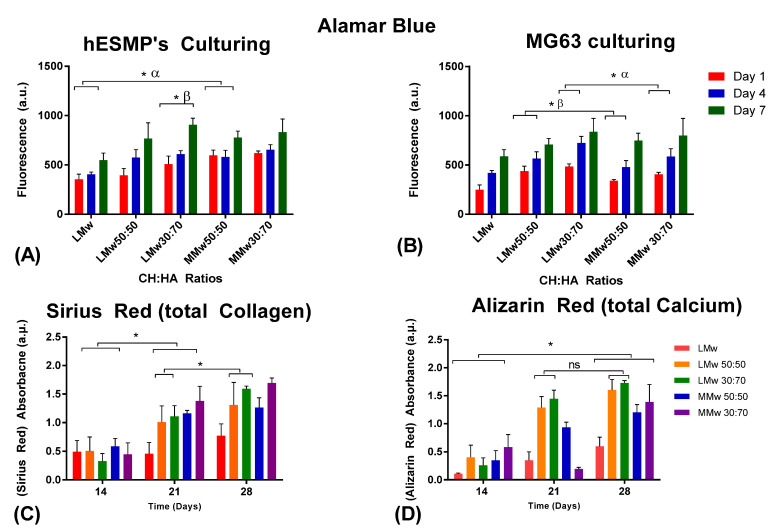
Cytotoxicity conducted via culturing (**A**) hES-MP and (**B)** MG63s. Cellular viability analysed with Alamar blue staining until day 7. Values shown are taken from mean ± SD where *n* = 3. (**C**) Collagen and (**D**) calcium accumulated by hES-MPs at day 14, 21 and 28. (ns, no statistically significant difference, α and β represent statistically significant difference in between the observed groups). (*) indicates statistically significant difference.

Cytotoxicity of the neat CH and composite membranes was assessed by culturing by hES-MPs and MG63s over a period of 7 days (Figure 7A,B). Cells were seeded on the side dominated by HA. All membranes showed initial attachment visible from day 1 values. LMw 30:70 displayed a steady increment, which was statistically significant from day 1 to 7. Cellular proliferation on HA loaded samples was higher when compared to neat LMw on day 7 of culturing hES-MPs. Similar ratios when seeded with MG63s show a steady increment for all specimens; however, greater values were observed for LMw 30:70 and MMw 50:50. A statistically significant difference was observed when MMw 30:70 was compared against LMw 30:70 in between day 1 and 7 values. To assess the mineralised matrix deposition, calcium and collagen production were quantified by conducting Sirius red and Alizarin red staining (Figure 7C,D). MMw 30:70 showed higher collagen deposition when compared with LMw, 50:50, 30:70 and MMw 50:50 at day 28.

## 4. Discussion

Amongst the other ideal properties of GTR membranes, one of the most pivotal function of it is to act as an occlusive barrier [20]. Results of optical images and SEM micrographs were indicative that the top surface was dominated by CH whereas the bottom surface had greater accumulation of HA. The membranes were hence visualised to have a bioinert interface on the CH side having absolutely no osteoconductivity (when facing soft tissue side). In addition, the HA side is the bioactive interface to trigger bone regeneration (facing the defected hard tissue side). Our study is the first to report on the high degree of seclusion achieved in between CH and HA. Clear discrepancies in the morphology can be seen on the top and bottom surface. A study performed by Li et al. showed that by enhancing the nano-HA (n-HA) concentration from 5% to 30%, surface irregularities also increased on either side of the membranes. Moreover, at 15%, rifts/fissures were also reported [27]. However, in our study, up to 70% HA addition was considered favourable, as an increment of HA will be an excellent candidate for its clinical application as a GTR membrane by creating a more osteoconductive membrane.

By reducing the speed at which solvent evaporates, we were able to achieve this high degree of seclusion that allowed HA to settle down due to the higher density by using solvent casting, which may be attributed to the difference in the surface free energy of HA and CH. This segregation was still enough to allow some interactions of HA to CH for bond formation as confirmed by spectroscopy. Spectroscopic findings were similar to the studies conducted previously that reported about HA interactions with CH [28,29].

Molecular interactions in between CH and HA have been discussed in detail in our previous study, which reports similar results [20]. Closer interpretations of the molecular pattern of LMw and MMw membranes have shown significant differences. The appearance of sharp intensity band at bands of –NH and –OH stretching vibrations cm^−1^ is indicative of hydroxyl band of HA interacting with CH [23]. Moreover, bands of –NH and –OH stretching vibrations between 3200 to 3400 cm^−1^ underwent shifts in the spectra from the bottom surface [20]. Thein et al. speculated that hydroxyl ions present on the surface of HA can interact with amino and hydroxyl groups on CH, thereby triggering the formation of hydrogen bonds [28]. With respect to the fingerprint region, the glycosidic bonds designated to CH that appear at 1153 cm^−1^ to 1015 cm^−1^ showed shifts in wavenumbers. Similarly, phosphate vibrations of υ_1_ at 960, υ_2_ at 1080, υ_3_ at 1096 and υ_4_ at 565, 603 and 472 cm^−1^ are seen in the spectral region obtained from the bottom surface of LMw and MMw 70:30 membrane. Investigations conducted on fabricating of CH:HA composite membranes with varying ratios of CH to HA have reported that the absorption bands around 1654 cm^−1^ and 1595 cm^−1^ shifted to lower wave numbers as HA amount increased [22,30]. This decrease in intensity of the HA band could be due to the gradual attachment among Ca^2+^ ions of HA to –NH_2_ groups of CH. This can be attributed to a possible covalent bond formation in between CH and HA [31].

The percentage of DD affects the overall mechanical properties of CH templates. Results of the tensile tests were indicative of the fact that addition of HA lowered the tensile strength. As reported by Teng and co-workers, a decrease in the tensile strength is because of the change in the crystallinity of the CH polymer matrix, leading to an alteration in the morphology of the membrane. Results obtained by Teng et al. were similar to the current study as they observed a rapid increment in *E* values as the HA amount increased, which was followed by a gradual decline [30]. Other reports about HA additions to polymers have shown a rise in the mechanical strength [32,33]. Since CH acts as a gel to hold HA together, less CH is available to hold HA together as the ratio varies in intensity, reducing the actual values of Young’s modulus. Abere and co-workers have reported 490 MPa E_appr_ values of 60:40 volume fraction for CH:HA and that of 30:70 to be 158 MPa. Furthermore, they mentioned a decrease in tensile strength values with increased reinforcement as HA acts as flaws in the polymer matrix as a result of poor interfacial bonding or decrease in force of adhesion between the HA matrix and CH [34]. Mechanical strength of templates affects the mechanotransduction of adherent cells [35]. Moreover, the regeneration process is affected by mechanical loading and stress distribution. A less rigid mechanical environment results in a prolonged bone regeneration phase [36]. Compared with other CH-based composite barrier membranes, the highly segregated membranes displayed better or comparable mechanical properties and retain adequate tensile strength to function as a barrier membrane [37,38]. It has been reported that the optimal scaffold stiffness for triggering bone regeneration could be significantly lower than that of the rigid bone [39]. Although there have been several reports on the interaction of CH:HA composite membranes, investigations on highly segregated membranes as reported in the current study warrant further in-depth analysis on the impact of these composite formulations and mechanical strength on cellular behaviour.

Cell biomaterials measurements are strongly reliant on wettability of biomaterials surface since these predict several biological factors such as cellular attachment, spread and proliferation [37]. Results of the swelling ratio of composite membranes were in agreement with investigations conducted previously [22,40]. Mohamed et al. reported that in composite membranes, the addition of HA reduced the swelling profile to 90% that had 40% CH [40]. This behaviour could be due to fact that hydroxyl ions of CH interact with hydroxyl groups of HA, resulting in a decline in the number of ions availability, which reduces the hydrophilic nature for composites [41]. The decrease in swelling ratio after 168 h in LMw membranes could be due to fact that after the passage of certain time degradation is triggered and swelling reaches an equilibrium, which is why a slight decline is visible. Mota et al. studied CH and Bioglass^®^ swelling properties and the results showed an increment in the graph after 42 days with a decline in the profile, which is similar to findings acquired in the present study [42]. Furthermore it was also reported that until 16 weeks, no changes were observed and after that, slow in vitro degradation may have been initiated [42]. Diffusion of water is rapid whilst penetrating into the CH ultrastructure hence, the matrix has the tendency to swell up before starting degradation. Another reason could be that the amorphous segments in the CH molecular structure could be more pervious to lysozymes penetration than the crystalline segments, and therefore, amorphous parts are prone to faster degradation than the crystalline segment [43,44]. Amongst a number of factors that govern CA on biomaterial surfaces, the microarchitectural features and chemical composition play a pivotal role. Although reports on the contact angle measurements of neat CH and composite HA formulations have disparity within the existing literature. Ranges from 83 to 113 [45,46] are indicative of hydrophobic surface whereas the bottom surface of highly segregated membranes had lower values, depicting a more hydrophilic surface [47,48]. This decrease in CA could be due to the irregular surface topography [47] or due to alterations in the chemical and physical ultrastructure, resulting in the availability of more hydroxyl groups from the addition of hydroxyapatite. The bottom surface of the membranes, depending on the concentration of HA, had voids and pores exhibiting hydrophilic nature due to the capillary effect on the liquid due to the pores [49].

Porous membranes show faster degradation when compared with thin films or membranes that are not porous. This is due to the ability of the degradation medium to attack two exposed surfaces of these kind of templates. This leads to a lesser weight loss of the membrane when they are compared to porous templates [50]. MMw membranes showed a more stable profile over the 48-day degradation period, which was similar to another study [51]. Tomihata et al. reported that biodegradation of such templates is not governed by the physical ultrastructure; rather, it is attributed to the chemical profile that is heavily influence by weight loss [52]. These variations in the weight of LMw 70:30, 50:50 and 30:70 could be due to a number of reasons. The enzyme must enter the membrane and react with the CH polymer. Pores provide a larger area for lysozyme to enter, with difference in the thickness having a significant effect on this [38]. One possibility could be that even thickness of the membranes leads to non-uniform rates among different ratios. CH chains are characterised as having sequences and lysozymes, containing six binding sites whereby there is preferential attachment of lysozymes to the CH units [53,54].

Tissue engineering templates made with CH alone and its composite formulations are known to be promising contenders for hard and soft tissue engineering applications amongst the other naturally occurring biopolymers. However, studies mentioning their biological performance report about the unpredictable and inconsistent nature of results. This could be due to the weak characterisation of such templates [55]. There is a strong correlation between cellular attachment and DD due to the cationic nature of CH undergoing attraction, which is in response to the amplification with DD. There is protonation of amine groups that are able to draw cells bearing a negative charge [55]. The attachment of MG63 on biomaterials is mainly reliant on fluctuations in the pH occurring during incubation period, due to adhesion kinetics. HA additions are able to contribute towards the macromolecular environment by balancing the pH, which is directly proportional to cellular adhesion and imparts cytocompatibility to the material. [55,56]. Another investigation carried out by Kong et al. mentioned that composite CH membranes were able triggered a greater rate of proliferation as compared to CH on its own [57].

Human osteosarcoma-derived cell lines, such as Saos-2, U-2 OS and MG-63, are often adapted to assess cytotoxicity of biomaterials. These cells are able to proliferate rapidly and indefinitely, and hence, are convenient in vitro cellular models [58,59]. On the other hand, mesenchymal stem cells such as hES-MPs are specific primary cells which are able to aid in determination of the osteoinductive properties of biomaterials. Furthermore, their culture on scaffolds targeting bone regeneration is also commonly reported [60]. Cell viability results were suggestive of the fact that membranes were supporting proliferation for both MG63s and hES-MPs from day 1 to 7. Although majority of the studies show that CH surface is conducive to osteoblast attachment and proliferation, CH template can restrict osteoblast proliferation and attachment [61]. The current investigation had similar findings, whereby CH was able to trigger initial attachment and viability, as well. These variations in the available literature could be due to the diverse nature of origin and biophysical properties of CH. Another relevant cause of the variations in cytotoxicity values can be assigned to the thickness of these membranes. Irregular thickness of these membranes is also known to play a vital role in the attachment of mesenchymal stem cells [62]. Since the cell cultures were performed on both LMw and MMw membranes, even the slightest degradation could lead to significant alterations in the overall film characteristics that may hinder the rate of cellular proliferation [55]. An important aspect of membrane technology is maintaining an even film thickness, especially for GTR membranes.

Membranes are able to stimulate higher levels of bone marker proteins and genes [39]. Collagen expression is a known marker for bone formation [63]. Studies conducted on assessing mineralised matrix deposition on CH and composite templates have reported that these biocomposites were not just able to trigger alkaline phosphatase (ALP) activity and collagen deposition but also elevate the expression of mRNA. This is designated as an osteogenic differentiation marker when cultured on bone marrow stromal cells (BMSCs) [63,64].

## 5. Conclusions

In the current investigation, we successfully engineered highly segregated CH/HA thin films in different amounts of HA with two varying molecular weights of CH to function as a surface layer of a spatially designed functionally graded membrane. A higher amount of HA was confirmed, which affected the physical and mechanical features of the template. Considering the time period reported as required for adequate regeneration of periodontal complex, it was found that LMwCH:HA with a ratio of 30:70 shows promising potential to serve as a functional gradient whilst fabricating a trilayered membrane. Solvent casting membranes layer by layer can also be adapted to achieve gradients to mimic natural tissue structure and function for future dental and biomedical applications.

## Data Availability

Not applicable.
